# Death of Dr. Leiper

**Published:** 1852-01

**Authors:** 


DIED,—On the 1st of December, 1851, after a brief illness, Dr. JAMES W. LEIPER, aged thirty-seven years.

Dr. Leiper was a member of the Northern Medical Association of Philadelphia, and by this afflicting dispensation the Society have lost an amiable and valued associate, and the community an able physician and exemplary citizen.

